# Sargramostim (rhu GM-CSF) as Cancer Therapy (Systematic Review) and An Immunomodulator. A Drug Before Its Time?

**DOI:** 10.3389/fimmu.2021.706186

**Published:** 2021-08-17

**Authors:** Hillard M. Lazarus, Carolyn E. Ragsdale, Robert Peter Gale, Gary H. Lyman

**Affiliations:** ^1^Department of Medicine, Case Western Reserve University, Cleveland, OH, United States; ^2^Medical Affairs, Partner Therapeutics, Inc., Lexington, MA, United States; ^3^Centre for Haematology, Department of Immunology and Inflammation, Imperial College London, London, United Kingdom; ^4^Public Health Sciences and Clinical Research Divisions, Fred Hutchinson Cancer Research Center, Seattle, WA, United States

**Keywords:** GM-CSF, sargramostim, immune modulation, innate immune response, adaptive immune response, granulocyte-macrophage colony-stimulating factor, immune therapy, cancer

## Abstract

**Background:**

Sargramostim [recombinant human granulocyte-macrophage colony-stimulating factor (rhu GM-CSF)] was approved by US FDA in 1991 to accelerate bone marrow recovery in diverse settings of bone marrow failure and is designated on the list of FDA Essential Medicines, Medical Countermeasures, and Critical Inputs. Other important biological activities including accelerating tissue repair and modulating host immunity to infection and cancer *via* the innate and adaptive immune systems are reported in pre-clinical models but incompletely studied in humans.

**Objective:**

Assess safety and efficacy of sargramostim in cancer and other diverse experimental and clinical settings.

**Methods and Results:**

We systematically reviewed PubMed, Cochrane and TRIP databases for clinical data on sargramostim in cancer. In a variety of settings, sargramostim after exposure to bone marrow-suppressing agents accelerated hematologic recovery resulting in fewer infections, less therapy-related toxicity and sometimes improved survival. As an immune modulator, sargramostim also enhanced anti-cancer responses in solid cancers when combined with conventional therapies, for example with immune checkpoint inhibitors and monoclonal antibodies.

**Conclusions:**

Sargramostim accelerates hematologic recovery in diverse clinical settings and enhances anti-cancer responses with a favorable safety profile. Uses other than in hematologic recovery are less-well studied; more data are needed on immune-enhancing benefits. We envision significantly expanded use of sargramostim in varied immune settings. Sargramostim has the potential to reverse the immune suppression associated with sepsis, trauma, acute respiratory distress syndrome (ARDS) and COVID-19. Further, sargramostim therapy has been promising in the adjuvant setting with vaccines and for anti-microbial-resistant infections and treating autoimmune pulmonary alveolar proteinosis and gastrointestinal, peripheral arterial and neuro-inflammatory diseases. It also may be useful as an adjuvant in anti-cancer immunotherapy.

## Highlights

- Sargramostim (yeast-derived rhu GM-CSF) accelerates bone marrow recovery after exposure to bone marrow damaging exposures. Safety and efficacy studies in other clinical settings warrant further study.
- Endogenous GM-CSF modulates the innate and adaptive immune systems and acts on multiple hematopoietic lineages.
- rhu GM-CSF and rhu G-CSF have distinct mechanisms of action with different clinical effects; they are not inter-changeable.
- Data in melanoma suggest the potential of adjuvant sargramostim to improve cancer outcomes and reduce the toxicity of immune checkpoint inhibitors.
- Sargramostim may have a role as an adjuvant to anti-fungal agents for resistant infections, improve immune suppression associated with sepsis and trauma, have benefit in lung disorders such as autoimmune pulmonary alveolar proteinosis and acute respiratory distress syndrome, and improve symptoms in neuro-degenerative disorders.

## Introduction

Granulocyte-macrophage colony-stimulating factor (GM-CSF) is a protein central to regulation of hematopoiesis in mammals (1). Mouse GM-CSF was molecularly-cloned followed by molecular cloning of human GM-CSF (2, 3). Subsequently, recombinant human (rhu) GM-CSF was developed as a drug, the most common and only FDA-approved form of which is sargramostim (Leukine^®^, Partner Therapeutics, Inc., Lexington, MA), which is also now designated on the list of FDA Essential Medicines, Medical Countermeasures, and Critical Inputs (4). Other rhu GM-CSFs include molgramostim and regramostim.

**Figure 1:**
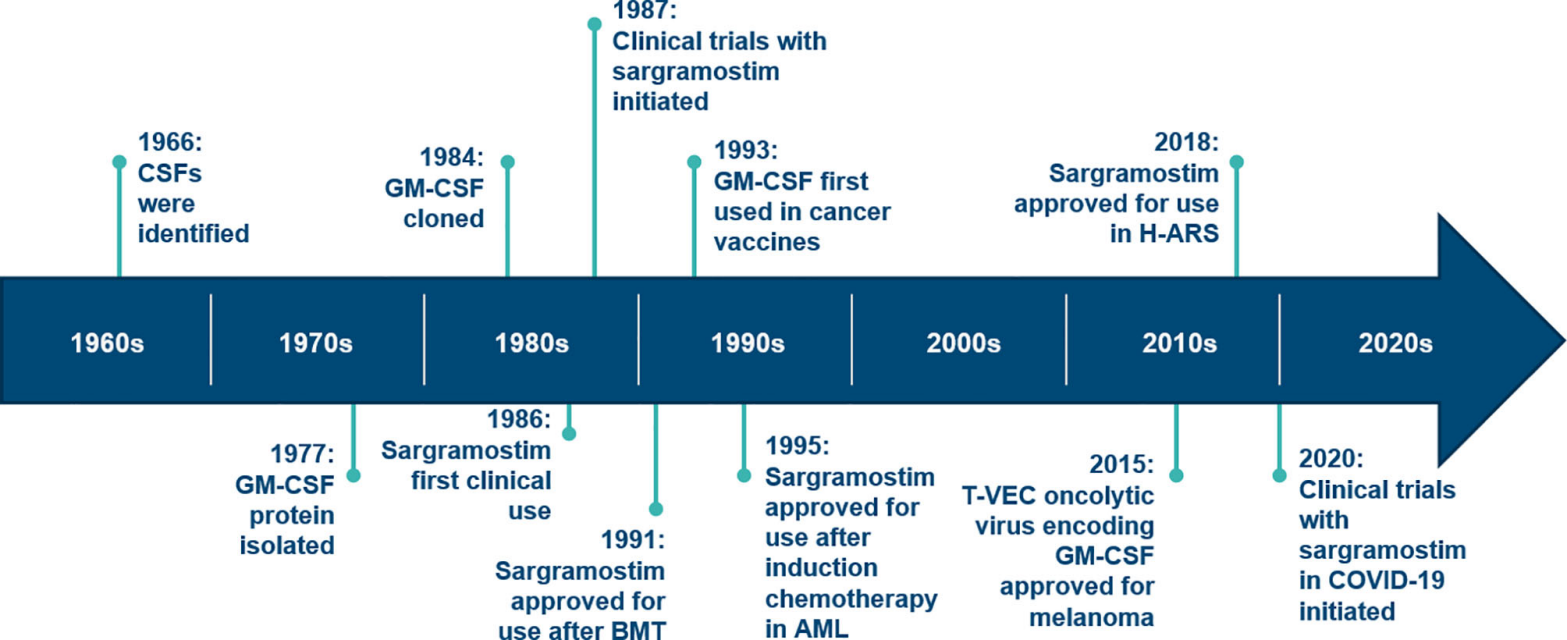
Timeline for discovery and development of GM-CSF. AML, acute myeloid leukemia; BMT, bone marrow transplant; CSF, colony-stimulating factor; GM-CSF, granulocyte-macrophage colony-stimulating factor; H-ARS, hematopoietic acute radiation syndrome; T-VEC, talimogene laherparepvec. (Modified from: Dougan M, Dranoff G, Dougan SK. GM-CSF, IL-3, and IL-5 family of cytokines: Regulators of Inflammation. Immunity. 2019;50(4):796‐811).

The development timeline of sargramostim, yeast-derived rhu GM-CSF, is displayed in **Figure 1**. The first clinical use of rhu GM-CSF was in 1986 when Gale and Vorobiov, in the aftermath of the Chernobyl nuclear power facility accident tested safety and efficacy of rhu GM-CSF by injecting themselves with a 10-fold higher dose than previously given to sub-human primates (5). Subsequently, these scientists used rhu GM-CSF to treat 3 radiation accident victims. Clinical trials began in 1987 and sargramostim was approved by US FDA in 1991 to accelerate bone marrow recovery in allogeneic hematopoietic cell transplant recipients (5, 6). It was subsequently approved in persons receiving bone marrow-suppressive drugs and/or radiation and in persons with post-transplant graft-failure. Although safe and effective in these settings, concerns over fever and myalgia resulted in a clinical shift to recombinant human granulocyte colony-stimulating factor (rhu G-CSF) in many clinical settings (7–11). Some believe this shift also may reflect effective marketing. In contrast, some animal and human studies suggest recombinant G-CSF is likely to exacerbate lung injury in the setting of infection *via* neutrophil infiltration whereas mice and humans receiving recombinant GM-CSF therapy may be associated with less lung injury risk [see review; Lazarus and Gale (12)]. Although the few comparative studies of sargramostim and rhu G-CSF products (filgrastim, pegfilgrastim and their biosimilars) report similar safety and efficacy, rhu G-CSF products account for over 95 percent of myeloid hematopoietic growth factor use (12–14). A formal comparison of the clinical uses of rhu GM-CSF *versus* rhu G-CSF is beyond the scope of this review [reviewed in Lazarus and Gale (12)].

We conducted a systematic review of clinical uses of sargramostim in cancer. Because sargramostim is the only approved rhu GM-CSF available for clinical use, we focus on this drug. In addition to the effects of sargramostim on accelerating bone marrow recovery, we describe use as an adjunct in host immune modulation, anti-cancer therapy, and as an anti-cancer vaccine adjuvant. We discuss why there is also renewed interest in sargramostim because of its pleiotropic biologic and immune-enhancing activities. The sargramostim US FDA approved indications and emerging uses as an immune modulator are detailed in **Table 1**.

**Table 1:** Labeled indications and investigational immunomodulatory uses for sargramostim.

Approved Indications^a^	Investigational Oncologic Uses	Investigational Non-oncologic Uses
*Hematopoiesis* Shorten time to neutrophil recovery and reduce incidence of severe and life-threatening infections and infections resulting in death following induction chemotherapy in adult patients with AML Mobilization of hematopoietic progenitor cells into peripheral blood for leukapheresis and autologous transplantation in adult patients Acceleration of myeloid reconstitution following autologous BMT or peripheral blood progenitor cell transplantation Acceleration of myeloid reconstitution following allogeneic BMT Treatment of delayed neutrophil recovery or graft failure after autologous or allogeneic BMT Increase survival in patients acutely exposed to myelosuppressive doses of radiation (H-ARS)	*Cancer Immunotherapy* In combination with immune checkpoint inhibitors In combination with other biologics (e.g., for neuroblastoma) Direct intra- or perilesional injection into tumors (cutaneous melanoma) *Vaccine Adjuvant* Tumor vaccines	*Infection* Adjunctive therapy for treatment of MDR refractory fungal and bacterial infections *Reversal of Immunoparalysis* Associated with critical illnesses (e.g., sepsis, MODS) *Respiratory Diseases* aPAP ARDS COVID-19 *Vaccine Adjuvant* Non-cancer vaccines (e.g., hepatitis B) *Neuro-degenerative Diseases* Parkinson disease Alzheimer disease *Peripheral Vascular Disease*

a Approved indications; see Leukine^®^ prescribing information for details (6).

AML, acute myeloid leukemia; aPAP, autoimmune pulmonary alveolar proteinosis; ARDS, acute respiratory distress syndrome; BMT, bone marrow transplantation; COVID-19, novel coronavirus 2019; H-ARS, hematopoietic syndrome of acute radiation syndrome; MDR, multidrug-resistant; MODS, multiple organ dysfunction syndrome.

## Methods

**Figure 2:**
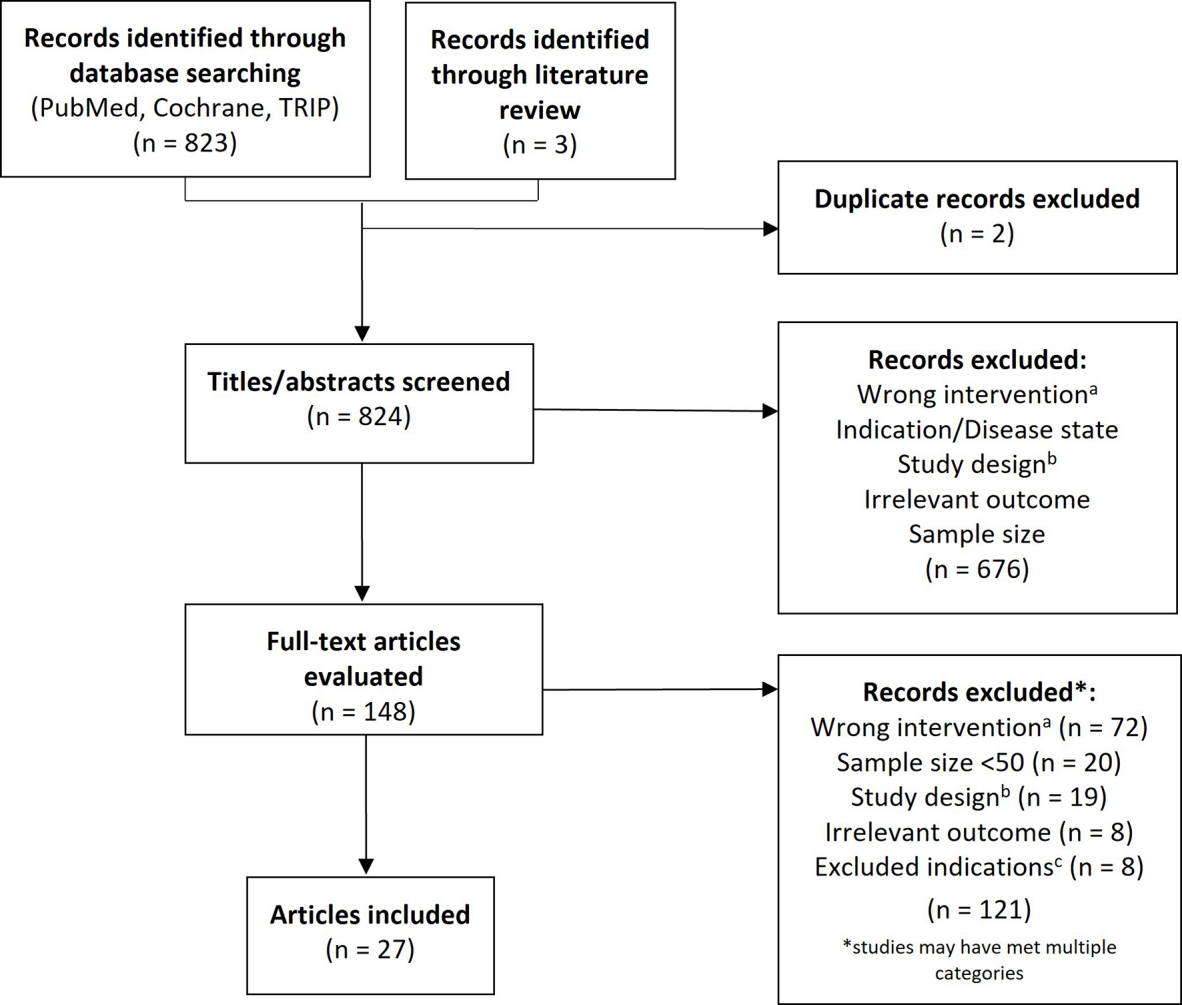
PRISMA flow diagram. PRISMA flow diagram. ^a^Intervention not sargramostim (e.g., molgramostim) and/or not administered *via* intravenous or subcutaneous route; ^b^Exclusions comprised phase 1 trials and those that were not prospective, interventional, or observational studies (e.g., letter to the editor, retrospective studies, etc.); ^c^Excluded studies were those assessing sargramostim for mobilization, as an adjuvant to vaccines or GM-CSF–expressing vaccines and studies supporting current FDA-approved indications.

This study was conducted according to the Preferred Reporting Items for Systematic Review and Meta-Analyses (PRISMA) guidelines (15) and followed a prespecified protocol. A literature search was performed in PubMed, Cochrane and TRIP databases (search terms: “*rhu-GM-CSF”* OR “*granulocyte-macrophage colony stimulating factor” OR “sargramostim”* AND “*cancer”*) to identify clinical studies published in English between January 1, 1988 and May 1, 2020 that evaluated the efficacy and/or safety of sargramostim in patients with hematologic or solid tumors (**Figure 2**). Additional studies found on a detailed literature review were also included. A two-stage approach was used in which three independent reviewers screened titles and abstracts followed by full-text articles based on pre-defined inclusion and exclusion criteria. Discordances were adjudicated and confirmed by the authors. Inclusion criteria encompassed prospective interventional or observational studies of sargramostim given IV or subcutaneously (SC), alone or combined with other cytokines and/or treatments with ≥ 50 subjects with cancer. We excluded studies assessing sargramostim for blood cells mobilization, as an adjuvant to vaccines or GM-CSF–expressing vaccines, alternate rhu GM-CSFs (*i.e.*, not sargramostim), those supporting current FDA-approved indications and all phase 1 studies. Data extracted included study-objective and -design, disease-type and, for clinical trials, safety and efficacy outcomes. All data were extracted and presented as reported in the original publications. Statistical significance, where specified, was based on the cut-off indicated in the original publication for determining significance (or *p* <.05 if not specified).

## Granulocyte-Macrophage Colony-Stimulating Factor

### Expression Systems

Sargramostim, produced in *Saccharomyces cervisae*, is a single-chain, glycosylated polypeptide (5, 6). It differs from human GM-CSF by substitution of leucine for arginine at position 23 rhu GM-CSFs derived in other expression systems [*i.e.*, molgramostim (bacterial-derived) and regramostim (mammalian-derived)] have been studied but are not commercially available. These rhu GM-CSFs are not inter-changeable because the expression system determines the amino acid structure and degree of glycosylation which in turn influences pharmacokinetics (distribution and clearance), biologic activity and safety (16, 17). Compared with other rhu GM-CSFs the intermediate level of glycosylation of sargramostim results in biologic activity like that of native GM-CSF, greater stability, resistance to degradation, improved tolerability and less immunogenicity. Several studies reported a lower frequency and severity of fevers, chills, myalgias, bone pain, edema, dyspnea and local erythema with sargramostim compared with *E. coli*-derived, non-glycosylated molgramostim (16, 18).

### Biology

GM-CSF causes rapid, sustained down-regulation and internalization of cell membrane GM-CSF receptor α subunit (GM-CSFRα) with the receptor playing a key role in ligand clearance (19, 20). Mice lacking a GM-CSF receptor develop high blood concentrations of GM-CSF after endotoxin challenge (21). Similarly, *loss-of-function* mutations in *CSFRA* is associated with markedly increased blood GM-CSF concentrations and pulmonary alveolar proteinosis because of the inability of alveolar macrophages to clear surfactant (22). Additionally, around birth there is a surge in alveolar GM-CSF causing immature alveolar macrophages to develop into functionally self-sustaining mature alveolar macrophages (23).

Wessendarp et al. recently reported the use of a GM-CSF receptor-β-chain deficient (Csf2rb–/–) murine model suggesting GM-CSF is critical for mitochondrial turnover, function and integrity (24). GM-CSF stimulation is required to maintain mitochondrial mass and function in macrophages, as well as for several other critical metabolic functions including self-renewal. Consequently, giving exogeneous GM-CSF may have profound effects on several metabolic pathways relevant to cellular proliferation including cytosolic and mitochondrial function.

### Actions

**Figure 3:**
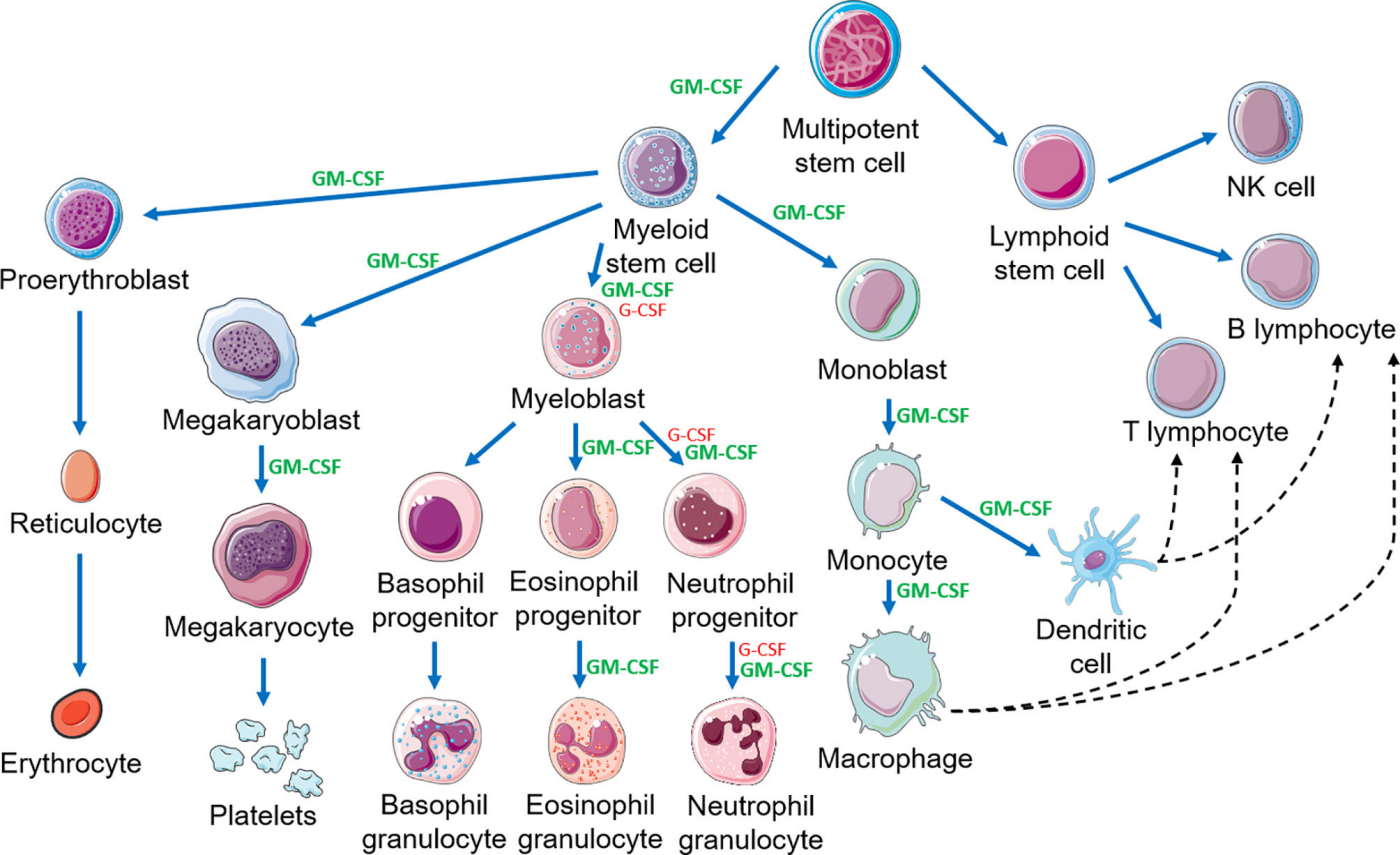
Hematopoietic cascade demonstrating the action of GM-CSF and G-CSF and the link between the innate and adaptive immune systems. This figure is a partial representation of the hematopoietic cascade. NK, natural killer. This figure was created using Servier Medical Art templates, which are licensed under a Creative Commons Attribution 3.0 Unported License; https://smart.servier.com.

**Figure 4:**
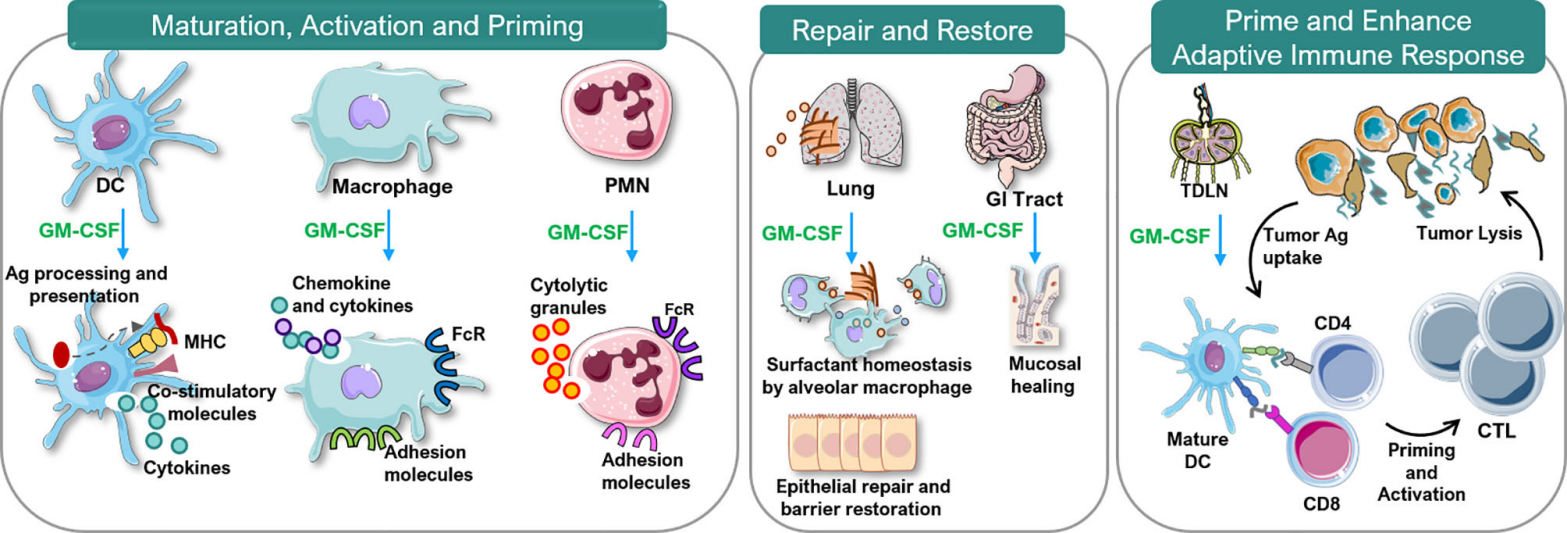
Immunobiology of GM-CSF. Ag, antigen; CTL, cytotoxic T lymphocytes; DC, dendritic cell; FcR, Fc receptor; GM-CSF, granulocyte-macrophage colony-stimulating factor; MHC, major histocompatibility complex; PMN, polymorphonuclear neutrophils; TDLN, tumor-draining lymph node. This figure was created using Servier Medical Art templates, which are licensed under a Creative Commons Attribution 3.0 Unported License; https://smart.servier.com.

Considerable data indicate GM-CSF stimulates proliferation, differentiation, activation and survival of granulocytes, monocytes and macrophages and stimulates growth and maturation of myeloid-derived dendritic cells (**Figure 3**) (1, 25–28). GM-CSF stimulates the innate immune response by activating macrophages and dendritic cells. By driving the immune function of dendritic cells, GM-CSF promotes development of antigen-specific T-cells and regulatory T-cells (Tregs), linking the innate and adaptive immune systems to increase host defenses (19). Additionally, cells associated with epithelial barriers, *e.g*. alveolar macrophages and gastrointestinal tract immune cells, are repaired and their functionality is restored by GM-CSF (**Figure 4**) (29, 30). Effects of GM-CSF on macrophages are thought to contribute to its ability to support epithelial cell function and repair of mucosal surfaces in the lung and gastrointestinal tract following direct injury or from infection (30, 31). **Figure 3** displays actions of GM-CSF and how it differs from granulocyte colony-stimulating factor (G-CSF) (12). Hence, GM-CSF increases immunity *via* the innate and adaptive immune systems.

#### Immune Modulation

**Figure 5:**
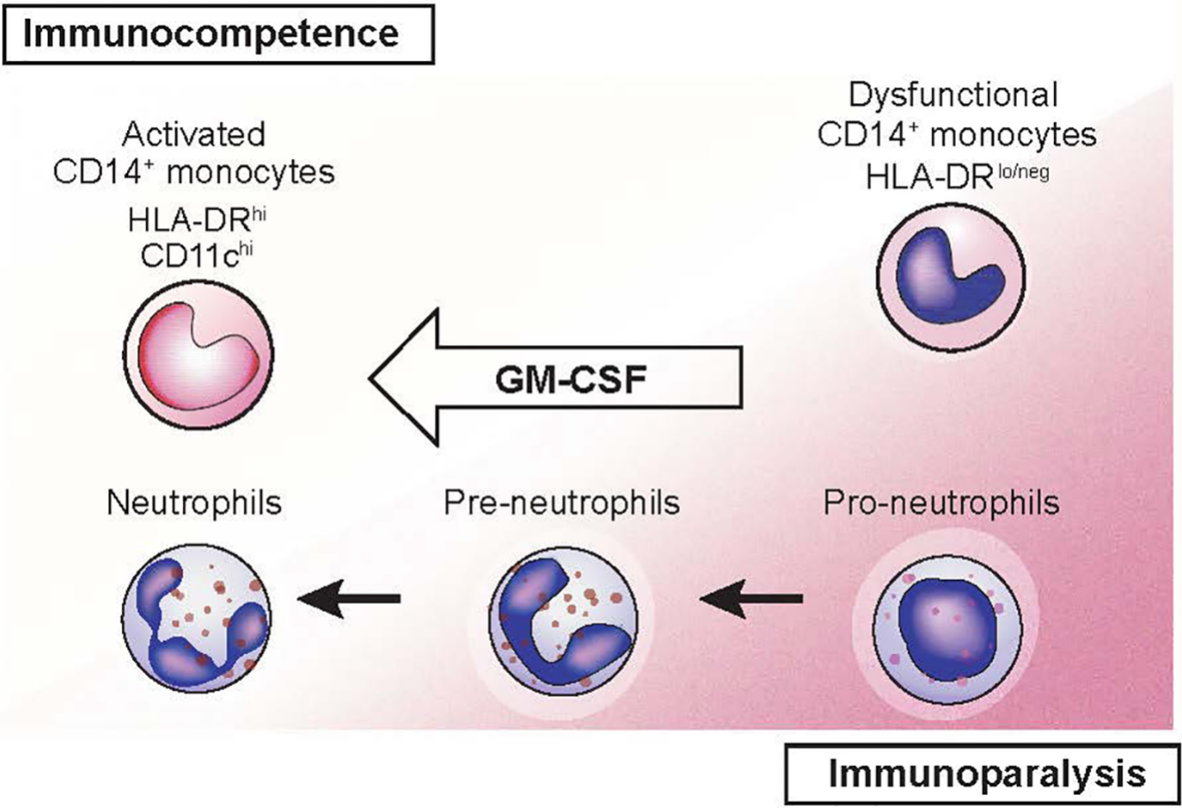
GM-CSF reversal of immune paralysis (modified from Schulte-Schrepping 2020).

GM-CSF increases monocyte killing of *S. aureus* and *C. albicans in vitro* in persons with solid cancers receiving chemotherapy (32). Sargramostim may reverse the immune suppression associated with severe illnesses such as sepsis, multiple organ dysfunction syndrome (MODS) and trauma (33–36). In persons with sepsis, sargramostim significantly increased the resolution rate of infections compared with placebo in a meta-analysis (37). In a randomized controlled trial of persons with severe sepsis or septic shock with sepsis-associated immune suppression, sargramostim increased monocyte HLA-DR levels, a marker of monocyte immune competence (38). Sargramostim-treated subjects had reduced need for mechanical ventilation and shorter intensive care unit and hospital stays. Hall et al. reported sargramostim prevented nosocomial infections in children with MODS (33). Considerable data indicate sargramostim stimulates immune function in persons with refractory bacterial and fungal infections including those with CARD9 deficiency (39–47). Recombinant GM-CSF is also reported to improve protection against and recovery from viral infections in mouse models (42, 48–50). Recent data indicate some cases of coronavirus disease-2019 (COVID-19)–associated acute respiratory distress syndrome (ARDS) results from cytokine release syndrome (CRS) (51–54). Sargramostim supports epithelial cell repair and reverses the immune suppression associated with CRS (31, 38, 55). Inhaled sargramostim could potentially reverse ARDS (56). Randomized trials testing this hypothesis in persons with COVID-19 are in progress (NCT04707664, NCT04326920, NCT04411680, NCT04642950) (57–61). **Figure 5** illustrates restoration of immune competence with GM-CSF from dysfunctional CD14^+^ HLA-DR^lo/neg^ monocytes to activated HLA-DR^hi^ monocytes (62, 63).

GM-CSF is typically characterized as a pro-inflammatory cytokine but some data suggest anti-inflammatory effects. Recent data indicate roles regulating the immune response (19). Sargramostim may be effective in several inflammatory, autoimmune and neuro-inflammatory diseases but convincing data from large randomized controlled trials are not reported (discussed in *Pulmonary and Gastrointestinal Repair*; *Emerging Uses in Neuro-degenerative Disorders*; *Other Emerging Uses*). 

#### Direct Anti-Tumor Properties of GM-CSF

GM-CSF stimulates production and proliferation of dendritic cells that may confer anti-tumor effect. Kurbacher et al. treated 19 subjects who had advanced cancers with sargramostim, 125-250 µg *per* day, subcutaneously (64). Subjects received a median of 4 prior drug regimens for metastatic breast, recurrent ovary, metastatic endometrial and recurrent cervix cancers. Sargramostim therapy was continued until progression or withdrawal of consent. Of the 19 subjects, one had a complete response and 6 had partial responses. Median response duration was 6 months. The authors attribute the anti-tumor response to dendritic cell activation by GM-CSF as well as GM-CSF-induced tumor growth arrest *via* stimulation of intratumoral GM-CSF receptors. The immune modulation driven by GM-CSF and the major role in the generation of dendritic cells from mononuclear precursors may be a signal as an important adjuvant in therapeutic vaccine approaches, which we discuss later (64).

#### Anti-Cancer Immune Modulation

Many cancers inhibit host immune responses facilitating cancer growth and metastasis, sometimes by inducing myeloid-derived suppressor cells (MDSCs), a heterogeneous population of immature myeloid cells which suppress innate and adaptive immunity (63). Monocytic MDSCs are monocytes with little or no HLA-DR expression and reduced antigen-presenting capability (CD14^+^ HLA-DR^lo/neg^ monocytes). In cancer MDSCs are important inhibitors of innate and adaptive anti-cancer immunity and decrease the efficacy of immune checkpoint inhibitors, CAR-T-cells and anti-cancer vaccines (65, 66). Some data suggest sargramostim can reverse this immune suppression by up-regulating HLA-DR expression and reversing effects of MDSCs and Tregs (33, 34, 38, 67–70). Up-regulation of monocyte HLA-DR expression and *ex vivo* lipopolysaccharide (LPS)-induced tumor necrosis factor-alpha responses are proposed biomarkers of immune suppression and could be useful to monitor responses to sargramostim therapy in persons with cancer (33, 34).

#### Anti-Cancer Vaccine Adjuvant

Sargramostim has been tested to enhance the efficacy of anti-cancer vaccines in several diseases. GM-CSF functions by recruiting and activating antigen-presenting cells at the injection site (71, 72). Cuzzubbo et al. reported sargramostim is the most common adjuvant in cancer vaccine trials (73). Results of these trials are mixed. Low doses of sargramostim, 40-80 μg for 1-5 days, may increase a vaccine-induced immune response, while higher doses may promote expansion of MDSCs (74). Petrina et al. reported adjuvant sargramostim in prostate cancer increased T-cell infiltration to the cancer microenvironment (75). The increased T cell infiltration into the tumor microenvironment may be promising for tumor suppression.

#### Pulmonary and Gastrointestinal Repair

GM-CSF facilitates repair of lung and gastrointestinal tract mucosa. In both tissues GM-CSF increases recruitment, differentiation and activation of dendritic cells and their interactions with T-cells to promote bacterial killing (30). GM-CSF also stimulates alveolar macrophage opsonization and phagocytosis of pathogens and enhances their clearance (31). GM-CSF promotes epithelial cell repair and prevents surfactant accumulation thereby supporting normal lung function. These observations prompted use of GM-CSF for pulmonary alveolar proteinosis (PAP).

PAP is a rare respiratory syndrome characterized by accumulation of surfactant in alveoli and terminal airways resulting in respiratory failure (76). In autoimmune PAP (aPAP), auto-antibodies to GM-CSF are found in broncho-alveolar lavage fluid and serum. PAP therapy with inhaled sargramostim appears to restore lung function and continues to be studied (77–80).

Paneth cells, highly specialized secretory epithelial cells located at the base of small intestinal crypts, express GM-CSF (81). Paneth and non-Paneth cells of the small intestine express the GM-CSFR β-chain. GM-CSF is also a key cytokine in the differentiation of intestinal dendritic cells and innate immune homeostasis (82). GM-CSF stimulates recruitment and activation of gut-derived immune cells, increases resistance to bacterial translocation and augments clearance of bacteria and viruses. In a murine model of Crohn disease GM-CSF activates monocytes and reduces disease severity (83). In humans, decreased bioavailability of GM-CSF is associated with more severe Crohn disease (84). Although most clinical data do not support a role for rhu GM-CSF in Crohn disease more studies appear needed (85).

### Comparison of GM-CSF and G-CSF

GM-CSF and G-CSF are the two most common hematopoietic growth factors. At times, they are thought to be interchangeable; however this is not true (12). GM-CSF is produced by B- and T-cells, granulocytes, monocytes, macrophages, fibroblasts, epithelial cells, endothelial cells and microglia (42, 86). The G-CSF receptor (G-CSFR) is primarily on granulocytes and myeloid bone marrow precursor cells whereas the GM-CSFR (CD116) is on granulocytes, monocytes, eosinophils, basophils, dendritic cells and possibly B-cells (19, 42, 87). GM-CSF binding to these receptors results in rapid internalization and activation of intra-cellular signaling. Consequently, GM-CSF stimulates production of granulocytes and macrophages from precursor cells in the hematopoietic cascade whereas G-CSF only stimulates production and activity of granulocytes (1, 88–90) (**Figure 3**). Different receptor distributions explain many differences in the biological activities of G-CSF and GM-CSF. In one study, rhu GM-CSF accelerated granulocyte recovery earlier compared to control but slower than rhu G-CSF in persons receiving bone marrow damaging drugs (13). Other reports indicate rhu GM-CSF stimulates immune responses in persons unresponsive to G-CSF (39, 43, 44, 91, 92). A retrospective analysis of persons with leukemia receiving a hematopoietic cell transplantation after high-dose pretransplant conditioning receiving colony stimulating factors suggested that receipt of rhu GM-CSF + rhu G-CSF was associated with greater cognitive improvement than rhu G-CSF alone (93). Randomized controlled trials of the effects of rhu GM-CSF on cognitive functioning in humans are warranted and underway to confirm these preliminary findings. As discussed, a critical comparison of clinical uses of rhu GM-CSF *versus* rhu G-CSF is beyond the scope of this review (reviewed in Lazarus and Gale, 2020) (12).

## Systematic Review

A total of 823 citations were screened by title and abstract of which 676 were excluded (**Figure 2**). Of the 148 articles included in the first-pass screening, 27 met all criteria for final inclusion following full-text screening and are detailed in **Tables 2** through **5** and **Supplementary Table 1**.

**Table 2:** Clinical studies evaluating immune-enhancing effects of sargramostim in melanoma.

Citation	Design/Patient Population	Treatment	Efficacy	Adverse Events	Comment
Spitler 2000 (94)	Phase 2; Multicenter; Open-label; Matched historical controls Melanoma (surgically-resected, stage III/IV; (N=96)	Sargramostim 125 µg/m^2^ SC d1-14 every 28d x 1 yr (or disease recurrence) *vs* matched historic controls	*Sargramostim vs historic controls:* • Median OS 37.5 mo *vs* 12.2 mo (p <.001) • DFS increased with sargramostim (p = .03)	*TRAE (sargramostim, all grades):* • Injection-site erythema 58% • Myalgias, weakness or fatigue alone or in combination 56% • Rash 10%	Increased OS and DFS with sargramostim
O’Day 2009 (95)	Phase 2; Single arm; Multicenter Melanoma (metastatic; chemotherapy-naïve without CNS metastases; 68% M1c; N=133)	Induction biochemotherapy: cisplatin, vinblastine, dacarbazine, IL-2, IFN alfa-2b and sargramostim 500 µg SC d6-15 (or beyond) until ANC >5,000/µL then: Maintenance biotherapy in responders (n=95) but only n=79 given sargramostim 250 µg/day on d1-14 of 28-d cycles	*Biochemotherapy + sargramostim:* • Induction response rate 44% (95% CI 35, 52) ○ CR 8% ○ PR 36% • Median PFS 9 mo (95% CI 8, 12) • Median OS 13.5 mo (95% CI 12, 15)	Hospitalizations for neutropenic fever: 3 *TEAE >10%, number patients with grade 3 and 4 (biochemotherapy + sargramostim):* • Leukopenia 38/32 • Thrombocytopenia 26/4 • Constitutional symptoms 21/3 • Cardiac 17/2 • Pulmonary 15/2	• No treatment-related or infection-related deaths • Authors attributed hypotension and capillary leak syndrome to IL-2 therapy
Spitler 2009 (96)	Phase 2; Single arm; Open label; Multicenter Melanoma (surgically resected, stage II [T4], III, or IV; N=98; 44% failed previous adjuvant therapy)	Sargramostim 125 µg/m^2^ SC d1-14 of 28-d cycle for 3 yr or disease recurrence	*Sargramostim:* • Median melanoma-specific survival not reached (median follow-up 5.3 yr) • 5-yr survival rate 60% (95% CI 49%, 70%) • Median DFS 1.4 yr (95% CI 1.0, 4.2) • Recurrence: 62 of 98 (63%) patients, 43 of 62 (69%) localized disease	*TRAE >10% (grade 1, 74% or grade 2, 8%)*: • Injection site reactions 68% • Fatigue 48% • Myalgias 10% *Serious TRAE* • Chest pain, n=1	Median melanoma-specific survival not yet reached (median follow-up of 5.3 yr)
Eroglu 2011 (97)	Phase 2; Single arm Melanoma (stage IV; N=52; 81% Stage M1c; brain metastases 21%)	Docetaxel and vinorelbine on d1 and sargramostim 250 µg/m^2^ SC on d2-12 of 14-d cycles	*Chemotherapy + sargramostim:* • 1-yr OS 48% • Median OS 11.4 mo (95% CI 190, 390 d) • PFS 4.8 mo (95% CI 91, 214 d)	*TRAE > 10% (grade 3-4):* • Neutropenia 31% • Anemia 15% • Febrile neutropenia 12%	Median OS 11.4 mo and PFS 4.8 mo
Hodi 2014 (98)	Phase 2; Randomized Melanoma (unresectable stage III/IV; N=245)	Ipilimumab 10 mg/kg IV d1 + sargramostim 250 µg SC on d1-14 of 21-d cycles *vs* Ipilimumab alone	*Ipilimumab + sargramostim vs ipilimumab alone:* • Median OS 17.5 mo *vs* 12.7 mo (p = .01) • Median PFS 3.1 mo *vs* 3.1 mo (p = NS)	*TRAE ipilimumab + sargramostim vs ipilimumab alone:* • Grade 3-5 overall AE 45% *vs* 58% (p = .04) • Grade 3-5 GI AE 16% *vs* 27% (p = .05) • Grade 3-5 pulmonary AE 0% *vs* 7.5% (p = .03)	• Increased OS with sargramostim • Decreased grade 3-5 overall, GI and pulmonary AE
Andtbacka 2015 (99)	Phase 3 Randomized; Open-label; Multicenter; Melanoma (unresectable stage IIIB-IV; N=436)	Sargramostim 125 µg/m^2^/day SC for 14 d in 28-d cycles *vs* T-VEC^a^ intralesional	*T-VEC^a^ vs sargramostim:* • Median treatment duration 23 wk *vs* 10 wk • Durable response rate 16.3% *vs* 2.1% (OR 8.9; 95% CI 2.7, 29.2; p <.001) • Overall response rate 26.4% *vs* 5.7% (p <.001) • Median OS 23.3 mo *vs* 18.9 mo (HR 0.79; 95% CI 0.62, 1.00; p = .051)	*TEAE T-VEC^a^ vs sargramostim:* • Injection-site erythema 5% *vs* 26% • Grade ≥3 AE 36% *vs* 21% (p = .003)	• OS favored T-VEC • Increased grade 3-4 TEAE with T-VEC • Injection-site erythema was the only AE that occurred at a higher incidence with sargramostim

a T-VEC (talimogene laherparepvec): herpes simplex virus type 1–derived oncolytic immunotherapy designed to selectively replicate within tumors and produce GM-CSF to enhance systemic antitumor immune responses.

AE, adverse event; CR, complete response; d, day(s); DFS, disease-free survival; GI, gastrointestinal; IFN, interferon; IV, intravenous; mo, month; OS, overall survival; PFS, progression-free survival; PR, partial response; SC, subcutaneous; TEAE, treatment-emergent adverse event; TRAE, treatment-related adverse event; NS, not significant; wk, week.

### Accelerating Hematopoietic Recovery

**Supplementary Table 2** displays the studies resulting in US FDA approval of sargramostim to accelerate bone marrow recovery in persons receiving bone marrow suppressing drugs and exposed to ionizing radiations (100–106). In persons with delayed post-transplant bone marrow function, sargramostim use led to neutrophil recovery within 14 days in a majority of patients and provided a survival advantage compared with historical controls (100). Giving sargramostim post-transplant accelerated the interval to neutrophil recovery and shortened hospital stay without increased toxicities compared with placebo (101, 103). In a 3-year follow-up study, long-term toxicities such as delay in bone marrow recovery, increased leukemia risk, or death were not increased in persons receiving sargramostim compared with placebo (102).

Sargramostim given to older adults with *de novo* acute myeloid leukemia (AML) during intensive induction and consolidation chemotherapy decreased treatment-related morbidity and mortality primarily related to fewer infections (104, 105). Lastly, sargramostim use improved survival of non-human primates following exposure to high doses of acute whole body ionizing radiations not receiving blood transfusions (106–108). Based on these data sargramostim is approved in several settings including: 1. shortening interval to neutrophil recovery and reducing incidence of severe and life-threatening infection following induction chemotherapy in AML; 2. accelerating myeloid reconstitution after autologous transplantation; 3. accelerating myeloid recovery following allogeneic hematopoietic cell transplantation; 4. therapy for post-transplant delayed neutrophil recovery or graft-failure; and 5. increasing survival after exposure to acute, high-dose ionizing radiations.

**Supplementary Table 1** displays data on effects of sargramostim on hematopoietic recovery after bone marrow suppressing exposures (7, 8, 10, 11, 13, 14, 109–113). Sargramostim was effective for enhancing neutrophil recovery in persons receiving cytotoxic therapy for non-Hodgkin lymphoma (NHL), plasma cell myeloma and breast cancer (7, 10, 110, 111).

Several studies compared efficacy of sargramostim and rhu G-CSF on neutrophil recovery in persons receiving bone marrow suppressive drugs. Beveridge et al. found no difference whereas Fields et al. reported delayed neutrophil recovery with sargramostim compared to rhu G-CSF therapy, but faster recovery compared to control (13, 14). Sargramostim *versus* no intervention was compared in two studies in subjects with small cell lung cancer (SCLC). Bunn et al. reported sargramostim was associated with less neutropenia but delayed platelet recovery and more non-hematologic adverse events and deaths (8). Steward and co-workers reported sargramostim failed to accelerate hematopoietic recovery nor decrease chemotherapy-related adverse events compared with placebo (112).

Overall, in a variety of settings, sargramostim administration after exposures to bone marrow suppressing agents usually accelerated hematologic recovery resulting in fewer infections, less therapy-related toxicity and sometimes improved survival.

### Anti-Cancer Immune Modulation

Studies of sargramostim in persons with cancer are displayed in **Tables 2** through 5. Anti-cancer effects of sargramostim were evaluated in 13 studies in solid cancers including melanoma (94–99) (**Table 2**), neuroblastoma (114–117) (**Table 3**) and prostate, colorectal and ovarian cancers (118–120) (**Table 4**). Anti-cancer effects of sargramostim were also evaluated in 3 studies in AML, chronic myelogenous (CML) and chronic lymphocytic leukemias (CLL) (121–123) (**Table 5**).

**Table 3:** Clinical studies evaluating immune-enhancing effects of sargramostim in neuroblastoma.

Citation	Design/Patient Population	Treatment	Efficacy	Adverse Events	Comment
Yu 2010 (114)	Phase 3; Randomized Neuroblastoma (newly diagnosed, high-risk with ≥PR after induction, myeloablative consolidation with hematopoietic cell rescue; N=226)	Post-consolidation therapy (28d cycles x 6): Standard chemotherapy: isotretinoin *vs* Immunotherapy + sargramostim: isotretinoin x 6 cycles + dinutuximab + alternating sargramostim and IL-2 x 5 cycles (sargramostim 250 µg/m^2^ x14d)	*Immunotherapy + sargramostim *vs* standard chemotherapy:* 2-yr event-free survival 66 ± 5% *vs* 46 ± 5% (p = .01) 2-yr OS 86 ± 4% *vs* 75 ± 5% (p = .02)	Grade 3-4 TEAE >10% *immunotherapy + sargramostim:* Neuropathic pain 52% Hypotension 18% Hypoxemia 18% Non-neutropenic fever 39% Infection 39% Catheter-related infection 13% Hypersensitivity reactions 25% Capillary leak syndrome 23% Urticaria 13% Diarrhea 13% Hyponatremia 23% Hypokalemia 35% Increase ALT 23% & AST 10%	Immunotherapy + sargramostim provided superior event-free survival and OS but greater incidence ≥ gr 3 AE
Cheung 2012 (115)	Phase 2; Single arm Neuroblastoma (disease status at study entry: primary/secondary refractory neuroblastoma; N=151)	Sargramostim 250 µg/m^2^ SC on d-5 to d1 then sargramostim 500 µg/m^2^ SC on d2-4 plus 3F8^a^ d0-d4 plus Isotretinoin added cycles 4-10	*Sargramostim + biochemotherapy:* CBRM1/5 (granulocyte activation marker) increased from 43.6% d0 to 67.2% d4 (p <.0001) Change in frequency and mean fluorescence intensity of CBRM1/5-positive granulocytes correlated with PFS (p = .024 and p = .008)	No AE reported	Sargramostim–induced granulocyte activation *in vivo* associated with improved patient outcome
		During cycle 4: Sargramostim given 250-500 µg/m^2^ IV d0-4			
Cheung 2014 (116)	Phase 2; Single arm	Sargramostim 250 µg/m^2^ SC x 5d then 3F8^a^ + sargramostim 250 µg/m^2^ SC x 2d then sargramostim 500 µg/m^2^ + 3F8^a^ x 3d (n = 79)	*Sargramostim + biotherapy *vs* historic control:* 5-yr PFS 24 ± 6% *vs* 11 ± 7% (p = .002) 5-yr OS 65 ± 6% 53% (of n = 40 MRD at enrollment) turned MRD negative after cycle 2 of sargramostim + biotherapy Overall complete remission: 3F8 + SC sargramostim: 68% 3F8 + IV sargramostim: 65%	*TEAE:* Grade 1-2 pain and urticaria	Increased PFS with SC *vs* IV sargramostim Correlation improvement in MRD with PFS
	Primary refractory neuroblastoma in bone marrow				
	Comparison historic control				
	Neuroblastoma (N=105)	Historic control with IV sargramostim (n = 26)			
Ozkaynak 2018 (117)	Phase 2; Single arm	Post-consolidation therapy (28d cycles): Immunotherapy + sargramostim: isotretinoin x 6 cycles + dinutuximab + alternating sargramostim 250 µg/m^2^/d SC or IV for 14d and IL-2 x 5 cycles	3-yr EFS 67.6 ± 4.8% 3-yr OS 79.1 ± 4.2%	Most common grade 3 or higher non-hematologic toxicities of immunotherapy were neuropathic pain, fever, hypotension, allergic reaction, capillary leak syndrome. *TEAE sargramostim *vs* IL-2:* No significant difference in allergic reactions, capillary leak syndrome and hypotension	AE generally resolved within 3d
	Neuroblastoma (newly- diagnosed, high-risk with ≥partial remission after induction, myeloablative consolidation with hematopoietic cell rescue; N=105)				

3F8 is an anti-GD2 monoclonal antibody.

AE, adverse events; d, day(s); EFS, event-free survival; IV, intravenous; IL-2, interleukin-2; MRD, minimal residual disease; OS, overall survival; PFS, progression-free survival; PR, partial response; SC, subcutaneous; TEAE, treatment-emergent adverse event; TRAE, treatment-related adverse event.

**Table 4:** Clinical studies evaluating immune-enhancing effects of sargramostim in prostate, colorectal and ovarian cancers.

Citation	Design/Patient Population	Treatment	Efficacy	Adverse Events	Comment
Aggarwal 2015 (118)	Phase 2; Randomized; Multicenter Prostate adenocarcinoma (metastatic castration resistant; N=125)	Maintenance sargramostim 250 µg/m^2a^ SC (500 µg^a^ maximum dose) on d15-28 of 28-d cycles *vs* observation	*Sargramostim vs observation:* • Median time to disease progression 3.3 mo *vs* 1.5 mo (p = .002) [post-hoc analysis as study not designed to compare outcomes between treatment arms] • PSA response recaptured in 2^nd^ chemotherapy course and given 2^nd^ maintenance: 62% *vs* 31% • Median OS 28.4 mo *vs* 14 mo	• 7 of 27 patients discontinued participation during sargramostim therapy • No treatment-associated deaths	Delayed time to disease progression with sargramostim
	Treatment with docetaxel + prednisone x 6 cycles with PSA response (≥50% decline): n = 52/125 (42%) randomized to maintenance				
Correale 2014 (119)	Phase 3; Randomized; Open-label; Multicenter	GOLFIG (includes sargramostim 100 µg SC d3–7) *vs* FOLFOX-4	*GOLFIG vs FOLFOX-4:* • Median PFS 9.2 mo *vs* 5.7 mo (HR 0.52; 95% CI 0.35, 0.77; p = .002) • Median OS 21.6 mo *vs* 14.6 mo (HR 0.79, 95% CI 0.52, 1.21; p = NS) • Response rate 66.1% *vs* 35% or 37.0%^b^ (p = .002) • Disease control rate 89.8% *vs* 61.7% or 64.8%^b^ (p = .001)	*TRAE >10% GOLFIG vs FOLFOX-4:* • Grade 2-3 hematologic 46% *vs* 34% • Diarrhea 19% *vs* 9% • Fever 19% *vs* 5% • Autoimmunity 19% *vs* 0% • Nausea/vomiting 15% *vs* 5% • Neurotoxicity 12% *vs* 5%	Increased PFS and OS with GOLFIG (sargramostim-containing regimen)
	Colorectal cancer (metastatic, chemotherapy-naïve; N=120)				
Schmeler 2009 (120)	Phase 2: Single arm Ovarian, fallopian tube and primary peritoneal cancer (recurrent, platinum-sensitive; N=59)	Carboplatin plus sargramostim 400-600 µg daily SC x two 7d courses (one preceding and one 24-36h after carboplatin) plus rIFN-γ1b 100 µg d5 & 7 of each 7d cycle of sargramostim	*Sargramostim + biochemotherapy:* • Overall response rate 56% • Median time to progression 6 mo	*TEAE >10%, grade 3-4:* • Fatigue: 28%/7% • Allergic reaction: 26%/0% • Neutropenia: 21%/7% • Thrombocytopenia: 16%/0% • Myalgia/arthralgias: 17%/0%	Response rate increased compared to other single-agent carboplatin trials

a Publication lists sargramostim dose as 250 mg/m^2^.

b Note discrepancy in published manuscript.

d, day(s); FOLFOX-4, 5-fluorouracil, levofolinate, oxaliplatin; GOLFIG, gemcitabine, oxaliplatin, levofolinate, 5-fluorouracil, IL-2, GM-CSF; mo, month(s); NS, not significant; OS, overall survival; PFS, progression-free survival; PSA, prostate-specific antigen; rIFN-γ1b, recombinant interferon, gamma 1b; SC, subcutaneous; TEAE, treatment-emergent adverse event; TRAE, treatment-related adverse event.

**Table 5:** Clinical studies evaluating immune-enhancing effects of sargramostim in hematologic malignancies (AML, CML, CLL).

Citation	Design/Patient Population	Treatment	Efficacy	Adverse Events	Comments
Rowe 2004 (121)	Phase 3; Randomized; Double- blind; Placebo-controlled; Multicenter AML (n=362)	Induction therapy plus priming with: Sargramostim 250 µg/m^2^ SC daily *vs* placebo SC daily starting 48h prior to induction:	*Sargramostim vs placebo priming study results:* • Complete remission 38% *vs* 40% (no p value) • No difference in induction therapy-related mortality, DFS and OS Complete remission rate in subjects not involved in priming was higher 50% *vs* 38% (p = .03)	Not reported	4- to 5-day delay in beginning induction chemotherapy due to sargramostim priming and randomization process
	Two randomizations: 1 - Induction therapy: daunorubicin, idarubicin, or mitoxantrone with cytarabine 2 - Priming: N=245 randomized to sargramostim or placebo	Then open label sargramostim 250 µg/m^2^/day SC until ANC ≥1500/µL x 3d, and 5d post-consolidation, sargramostim 250 µg/m^2^/day SC until ANC ≥1,500/µL for 3d			
Cortes 2011 (122)	Phase 2; Randomized CML (<12 mo from diagnosis, Philadelphia-chromosome positive, chronic phase, n=94)	High-dose imatinib x 6 months, then PEG IFN α-2b + sargramostim 125 µg/m^2^ thrice weekly + high-dose imatinib *vs* high-dose imatinib alone	*Sargramostim vs no sargramostim group:* No difference in complete cytogenetic response, major and complete molecular response, PFS, event-free and OS	Sargramostim discontinued in all patients due to TEAE	Increased AE in sargramostim + PEG-IFN + high-dose imatinib compared to high-dose imatinib alone necessitated discontinuation sargramostim limiting study conclusions
Strati 2014 (123)	Phase 2; Single arm CLL (frontline, n = 60) *vs* n = 166 historic control of FCR-alone (without sargramostim)	FCR plus sargramostim 250 µg/m^2^ SC on d −1 and d5–11 of course 1 and on d −1 and d4–10 of courses 2–6	*Sargramostim + chemotherapy:* • Overall response rate 100% • At median 56 mo follow-up, event-free and OS not reached FCR + sargramostim *vs* historic comparison FCR alone showed higher partial remission rate (p = .03)	• Grade 3-4 neutropenia 83% • Grade 3-4 infections 16% • Discontinuations 18%	Compared to historic controls, fewer infections with addition sargramostim, 15% *vs* 28% (p = .05)

AE, adverse event; AML, acute myeloid leukemia; ANC, absolute neutrophil count; CLL, chronic lymphocytic leukemia; CML; chronic myelogenous leukemia; d, day(s); DFS, disease-free survival; FCR, fludarabine, cyclophosphamide, rituximab; mo, month(s); OS, overall survival; PEG IFN α-2b, peglyated interferon alpha-2b; PFS, progression-free survival; SC, subcutaneous; TEAE, treatment-emergent adverse event.

#### Melanoma

Adding sargramostim to conventional melanoma therapies improved outcomes in some studies (**Table 2**). Hodi et al. (98) reported increased survival when sargramostim was added to immune checkpoint inhibitor (ipilimumab 10 mg/kg) treatment in a randomized phase 2 trial. Overall grade 3-5 treatment-related adverse effects were reduced including fewer gastrointestinal and no grade 3-5 pulmonary toxicities. Spitler and co-workers (94) reported increased disease-free and overall survival in subjects receiving sargramostim compared to matched historical controls. A study by Andtbacka et al. reported subcutaneous sargramostim and intra-lesional T-VEC therapy (a herpes simplex virus type 1–derived oncolytic immune therapy designed to selectively replicate within cancers and produce GM-CSF to enhance systemic antitumor immune responses) had similar survival rates in a phase 3 trial, ultimately leading to FDA approval for the use of T-VEC in melanoma (99). In a phase 2 study Spitler et al. (96) gave GM-CSF maintenance therapy to subjects with high risk of recurrence. Melanoma-specific survival was not reached after a median of 5.3 years follow-up, longer than observed in previous melanoma trials (**Table 2**). Further encouraging results are reported with the addition of sargramostim to other chemo- and bio-therapies (95, 97). In most of these studies treatment-related adverse effects of sargramostim were mild, the most common being injection-site erythema and fatigue (94–96, 98, 99), with the potential for a reduction in adverse effects with the addition sargramostim to therapy (98).

Several studies with sargramostim in persons with metastatic melanoma did not meet the literature search inclusion criteria. Si et al. and Elias et al. reported that intra-lesional injection of sargramostim in persons with metastatic melanoma resulted in regression of both injected and non-injected lesions suggesting systemic immune effects (124, 125). Additionally, reduced adverse events with the combination of ipilimumab 3 mg/kg (126) and 10 mg/kg (127) and sargramostim were reported in two *real-world* studies that corroborate the data presented here.

##### ICOS Ligand

Inducible co-stimulatory molecule ligand (ICOS-L) is a protein expressed on antigen-presenting cells, B- and T-memory and -effector cells, macrophages and dendritic cells (128) and on cells in the cancer micro-environment. Inhibition of the ICOS/ICOS-L signaling pathway on melanoma cells could improve treatment outcomes (129). Ipilimumab, a CTLA-4 inhibitor, increases CD4^+^ and CD8^+^ T-cell numbers and ICOS expression (130). Hodi and co-workers reported sargramostim added to ipilimumab increased ICOS expression on CD4^+^ and CD8^+^ T-cells compared with ipilimumab only (p = .11 and p = .01, respectively) (98) suggesting potential synergism.

#### Neuroblastoma

Sargramostim combined with an anti-GD2 monoclonal antibody was evaluated predominately in children with neuroblastoma (**Table 3**) (114–117). Yu and colleagues and Cheung and colleagues reported improved progression-free survival (PFS) and survival in subjects receiving sargramostim-containing regimens compared to standard chemotherapy and historical controls, respectively (114, 116). Adverse events were manageable. Based on these data dinutuximab (anti-GD2 monoclonal antibody, Unituxin^®^, United Therapeutics, Corp.) received FDA approval for the treatment of some children with high-risk neuroblastoma combined with rhu GM-CSF (*i.e.*, sargramostim), IL-2 and isotretinoin (131). Naxitamab-gqgk (Danyelza^®^, Y-mAbs Therapeutics, Inc.), a humanized GD2-binding monoclonal antibody, also received FDA approval in combination with rhu GM-CSF (*i.e.*, sargramostim) in certain patients with high-risk neuroblastoma (132).

##### Neutrophil-Driven Antibody-Dependent Cell-Mediated Cytotoxicity in Neuroblastoma

Sargramostim enhances neutrophil-driven antibody-dependent cell-mediated cytotoxicity (ADCC) in neuroblastoma when given with anti-GD2 antibodies (133). Patients with neuroblastoma undergoing high-dose chemotherapy have significantly attenuated lymphocyte anti-tumor responses, but neutrophils and macrophages are only transiently suppressed and can exert cell-mediated cytotoxicity and ADCC for marked tumor cell destruction (133, 134). Furthermore, the relative lack of complement-inhibitory proteins on the neuroblastoma cell surface renders these cancer cells more susceptible to cell-mediated cytotoxicity and antibody dependent cell cytotoxicity (135). Kushner and colleagues reported a 31 subject phase 1 dose-escalation study of anti-GD2 antibody therapy with sargramostim in treatment-resistant neuroblastoma (136). Fourteen (45%) subjects had complete or partial remission.

#### Other Solid Cancers

Adding sargramostim to conventional therapies was studied in other cancers including prostate, colorectal and ovary cancers (**Table 4**) (118–120). Investigators reported higher response rates with sargramostim and sargramostim-containing regimens compared with other similar trials. None of these studies were adequately controlled making a definitive conclusion impossible. These data suggest sargramostim may enhance anti-cancer responses in solid cancers when combined with conventional therapies but needs confirmation.

#### Hematologic Cancers

A few studies on the use of sargramostim in hematologic malignancies were included in this systematic review (**Table 5**). Some data suggest sargramostim might improve outcomes and reduce infections in persons with CLL receiving rituximab (123). In a study in subjects with CML adding sargramostim was no better than conventional therapy (122). Rowe et al. reported a phase 3 study in previously untreated persons with AML (121). Subjects received sargramostim before induction chemotherapy to stimulate leukemia cells to divide and thereby increasing their sensitivity to cell-cycle active drugs. No benefit was reported (121). In a recent report, Rong and associates (137) used an animal model to explore why some persons with extra-nodal natural killer/T cell lymphoma have rapid disease progression with GM-CSF treatment. They reported GM-CSF facilitated immune evasion by up-regulating PD-L1 expression. This effect could paradoxically increase immune checkpoint inhibitor activity similar to the use of prednisolone to increase CD20 cell expression and increase sensitivity to rituximab in children with B-cell precursor acute lymphoblastic leukemia during induction chemotherapy (138).

### Attenuation of Adverse Events

**Table 6** summarizes the evidence for sargramostim attenuation of adverse events when given with concurrent treatments in trials involving melanoma, AML and hematopoietic cell transplants. Reductions in infections and fatal infection are reported (100, 101, 103, 104). Decreases in other adverse events included less grade 3-5 AEs, grade 3-5 gastrointestinal (GI) and pulmonary AEs and mucositis (98, 103).

**Table 6:** Sargramostim attenuation of adverse events of concurrent treatments.

Citation	Patient Population	N	Sargramostim Treatment	Adverse Events (Sargramostim Arm *vs* Comparator Arm)	*P* Value
Hodi 2014 (98)	Melanoma (unresectable stage III/IV)	245	Ipilimumab + sargramostim 250 µg/d SC d1-14 of each 21-d cycle *vs* Ipilimumab alone	• Grade 3-5 overall adverse events: 45% *vs* 58% • Grade 3-5 GI adverse events 16% *vs* 27% • Grade 3-5 pulmonary adverse events 0% *vs* 7.5%	• 0.04 • 0.05 • 0.03
Rowe 1995 (104); Rowe 1996 (80)	AML undergoing induction chemotherapy with daunorubicin and cytosine arabinoside	124	250 µg/m^2^/day IV over 4 hr until ANC ≥ 1500/µL x 3d (consecutive) or for maximum of 42d *vs* Placebo	• Death from infection: 6% *vs* 23% • Death from fungal infection (overall): 2% *vs* 19% • Death from grade 3-4 fungal infection: 13% *vs* 75% • Death related to grade 3-4 pneumonia (among patients with pneumonia): 14% *vs* 54%	• 0.019 • 0.006 • 0.02 • 0.046
Nemunaitis 1995 (103)	Allogeneic BMT for various lymphoid neoplasias	109	250 µg/m^2^/day IV over 4 hr x 20d *vs* Placebo	• Infection rate: 64% *vs* 91% • Bacteremia: 17% *vs* 34% • Grade 3-4 mucositis: 8% *vs* 29%	• 0.001 • 0.043 • 0.005
Nemunaitis 1991 (101)	Autologous BMT for various lymphoid neoplasias	128	250 µg/m^2^/day IV over 2 hr x 21d *vs* Placebo	• Infection during first 28d: 17% *vs* 30%	• NS^a^
Nemunaitis 1990 (100)	Graft failure following BMT for cancer or aplastic anemia	37	60–1000 µg/m^2^/day IV over 2 hr x 14–21d *vs* Historic controls	• Death rate due to infection: 21% *vs* 59%	• NR

a Only 3.1% of sargramostim-treated patients had infections other than with streptococcus compared with 19.0% of placebo patients (P = 0.004). On the sargramostim arm the only bacterial infection was streptococcal bacteremia, whereas multiple pathogens (streptococcal, staphylococcal, fuso-bacterium and Corynebacterium bacteremia; staphylococcal cellulitis; legionella pneumonia) were detected in patients on the placebo arm.

AML, acute myelogenous leukemia; ANC, absolute neutrophil count; BMT, bone marrow transplantation; d, day(s); GI, gastrointestinal; hr, hour; IV, intravenous; NR, not reported; NS, not significant; SC, subcutaneous.

### Sargramostim Enhancement of Abscopal Response

Radiation therapy has not been thought to elicit an immunologic effect, but there are several reports of inducing a clinical immune response in a cancer site non-contiguous with the radiation field. This phenomenon is referred to as abscopal effect. Leary and colleagues reviewed clinical trials and case reports where sargramostim was added to radiation therapy (139). They discuss enhancement of the abscopal effect and postulate that sargramostim enables the presentation of tumor-associated antigens to generate a T-cell response. Golden and co-workers reported a phase 2 trial in 41 subjects with diverse cancers (140). Abscopal responses were observed in 4 of 18 patients with lung cancer, 5 of 14 with breast cancer and 2 of 2 with a thymoma. These responses were associated with improved overall survival. Leary et al. also discuss abscopal effects with sargramostim and radiation therapy in 2 persons with pancreatic and lung cancers (139).

## Discussion and Future Considerations

The sargramostim studies spanning 30 years of use highlight the extensive knowledge of sargramostim as a myeloid hematopoietic growth factor in accelerating bone marrow recovery after insult from bone marrow damaging exposures. The data presented in this systematic review suggest the innate and adaptive immune activity of sargramostim may improve cancer outcomes and reduce toxicity of chemo- and other immune therapies, including immune checkpoint inhibitors and GD2-binding monoclonal antibodies. Safety and efficacy in oncologic clinical settings warrants further study. Sargramostim may be less efficacious in hematologic malignancies, although data are limited.

The use of sargramostim in combination with immune checkpoint inhibitors in melanoma merits further study, as demonstrated by significantly mitigating immune checkpoint inhibitor-related adverse events. In melanoma, data indicate sargramostim improves survival and reduces toxicity associated with ipilimumab 10 mg/kg therapy (98). Furthermore, sargramostim is actively being studied in combination with the immune checkpoint inhibitor combination ipilimumab and nivolumab in patients with advanced melanoma; the ECOG phase 2-3 study achieved the phase 2 metric allowing the continuation into phase 3 (141). Also, sargramostim in combination with pembrolizumab is being studied in advanced melanoma (142). Other solid tumor investigations include sargramostim and pembrolizumab in biliary cancer (143, 144). As noted above, two GD2-binding monoclonal antibodies, dinutuximab and naxitamab-gqgk, are approved for use in combination with sargramostim in neuroblastoma (131, 132, 145).

Diverse activities of sargramostim are being evaluated (**Table 1**). These studies focus on increasing host defenses, reversing immune suppression, as a vaccine adjuvant and as combination therapy with immune checkpoint inhibitors. Other studies are evaluating different routes of administration such as intra- and peri-lesional injections for skin cancers. Many clinical trials evaluating the potential of sargramostim as an adjuvant for anti-cancer vaccines in diverse settings and improving efficacy of anti-bacterial and -virus vaccines are discussed above (146, 147).

### Emerging Uses in Neuro-Degenerative Disorders

GM-CSF treatment is protective in animal models of neuro-degenerative disorders (148–150). Increasing Treg activity is postulated to improve signs and symptoms of disorders such as Alzheimer and Parkinson diseases, amyotrophic lateral sclerosis and stroke (151). Sargramostim is being evaluated in Parkinson disease (152) based on *in vitro* and recent pre-clinical studies reporting a positive influence on innate and/or adaptive cell-mediated immunity. The focus has been on regulatory T-cells (Tregs), a T-cell subset which modulates the immune system, maintains tolerance to self-antigens, prevents autoimmunity and downregulates induction and proliferation of effector T-cells. GM-CSF increases the frequency of Tregs suggesting increased immune regulation and efficacy in Parkinson disease (153, 154). The anti-inflammatory activity of sargramostim appears mediated by inducing tolerogenic dendritic cells and thereby preventing T-cell activation (84, 155, 156). Olson and colleagues (157) reported sargramostim, 3 μg/kg/day, given for one year was well-tolerated and increased numbers and function of Tregs in persons with Parkinson disease. Sargramostim supported a neuro-protective biomarker phenotype associated with stable Parkinson disease.

Potter and associates followed their preclinical murine model (158) by a randomized, double-blind, placebo-controlled clinical trial in patients with mild-to-moderate Alzheimer disease (AD) (159). Forty patients were given either placebo or sargramostim 250 µg/m^2^/day subcutaneous injection five days/week for 3 weeks; compared to placebo and baseline, sargramostim recipients showed significant improvement in Mini-Mental State Examination (MMSE) scores, along with a decrease in plasma markers of neurodegeneration, tau and plasma ubiquitin C-terminal hydrolase L1 (UCH-L1), and an increase in amyloid beta (Aβ40), an amyloid marker that is decreased in AD (159). Clinical studies with sargramostim therapy in neuro-inflammatory disease continue.

### Other Emerging Uses

Other potential uses of sargramostim include as an adjunctive therapy in refractory bacterial and fungal infections and to reverse immune deficiency associated with sepsis and other acute illnesses (42, 47). Sargramostim mobilizes progenitor cells, particularly those of endothelial origin which are involved in vascular repair and regeneration into the circulation. These cells contribute to neo-vascularization in persons with symptomatic peripheral arterial disease (claudication) in whom sargramostim may improve walking performance (160, 161).

Lastly, as discussed above, sargramostim may be effective in respiratory diseases including autoimmune PAP (77–80) and the immune suppression associated with ARDS (38). There are ongoing trials of whether inhaled sargramostim might be effective in lung diseases such as COVID-19 (NCT0470766, NCT04326920, NCT04411680, NCT04642950) (57–60). The sum of these studies suggests a much wider potential role for sargramostim therapy than simply accelerating bone marrow recovery.

## Conclusions

GM-CSF accelerates hematopoietic recovery after exposure to bone marrow-suppressing agents and reverses post-transplant graft-failure. In this systematic review we consider use of sargramostim in other settings including therapy of solid cancers as an immune modulating drug. The favorable clinical outcomes observed in patients treated with sargramostim, combined with its acceptable safety profile and diverse biological effects, warrant continued evaluation of its role as immunotherapy. Furthermore, we describe use as an adjunct to therapy for resistant infections, immune suppression associated with sepsis and trauma, and respiratory and neuro-inflammatory diseases. We emphasize the pleiotropic biologic activities of GM-CSF including effects on innate and adaptive immune responses. These considerations and encouraging results of exploratory clinical trials suggest continued evaluation of this agent in diverse conditions.

## Research Agenda

- Evaluate the ability of sargramostim to restore immune function.
- Evaluate sargramostim in the setting of immune checkpoint inhibitor therapy.
- Develop new, targeted ways to administer sargramostim including delivery as an inhalation.
- Evaluate sargramostim in infection and as a vaccine adjuvant.
- Explore sargramostim in the treatment of peripheral artery disease and neuro-inflammatory disease.

## Data Availability Statement

The original contributions presented in the study are included in the article/**Supplementary Material**. Further inquiries can be directed to the corresponding author.

## Author Contributions

GL and CR designed the systematic review. CR, along with two other data abstractors, conducted the systematic review. HL, CR, RG, and GL summarized the data and prepared the typescript. All authors contributed to the article and approved the submitted version.

## Funding

Publication of this manuscript was supported, in part, by Partner Therapeutics, Inc.

## Conflict of Interest

HL is a paid consultant for Partner Therapeutics, Inc., including in relation to this manuscript. HL has stock options and participated on an advisory board for Partner Therapeutics, Inc. outside of the current work. CR is an employee of and has stock options for Partner Therapeutics, Inc. RG and GL received funds from Partner Therapeutics for consulting but none in relation to this manuscript. Additionally, RG is a consultant to BeiGene Ltd., Kite Pharma, Inc., FFF Enterprises, Inc., CStone Pharmaceuticals; advisor to LaJolla NanoMedical, Inc, Mindsight Pharma, Antegene Biotech LLC; partner, AZAC Inc.; Board of Directors for Russian Foundation for Cancer Research Support; and Scientific Advisory Board for StemRad Ltd. Additionally, GL is PI on research grant from Amgen to the Fred Hutchinson Cancer Research Center and has consulted for G1 Therapeutics, BeyondSpring, Sandoz, ER Squibb, Seattle Genetics, Jazz Pharmaceuticals, TEVA, Samsung, and Merck outside of the current work.

The authors declare that this study received funding from Partner Therapeutics, Inc. The funder had the following involvement with the study: Partner Therapeutics funded the professional writer, Jill Luer (Secundum Artem) who contributed to study design and data abstraction, and another data abstractor.

## Publisher’s Note

All claims expressed in this article are solely those of the authors and do not necessarily represent those of their affiliated organizations, or those of the publisher, the editors and the reviewers. Any product that may be evaluated in this article, or claim that may be made by its manufacturer, is not guaranteed or endorsed by the publisher.
