# Supplementary material for: Sargramostim (rhu GM-CSF) as Cancer Therapy (Systematic Review) and An Immunomodulator. A Drug Before Its Time?
Source: Front Immunol. 2021 Aug 17;12:706186. doi: 10.3389/fimmu.2021.706186 (PMC8416151; doi:10.3389/fimmu.2021.706186)
Supplement: Supplementary file 1 [file Table_1.pdf]

Supplementary Material

Table 1. Studies evaluating sargramostim for hematopoietic recovery after myelosuppressive therapy

Citation	Design/Patient Population	Treatment	Efficacy	Treatment Adverse Events Specific to Sargramostim or Comparator	Comment
Fields 1994 (1)	Phase 2<br><br>Hematologic (n=22; lymphoma: n=19) and solid malignancy (n=93; breast cancer n=80)	ICE then ABMT;<br>Sargramostim 250 µg/m²/d IV over 2h (max 21d) (n=47)<br>vs<br>G-CSF 10 µg/kg/d IV over 30min (n=24)<br>vs<br>no CSF (n=36)	*Sargramostim vs G-CSF vs no CSF:*<br>• Median time to ANC >500/µL: 18d vs 16d vs 21d (p = .001[b])<br>• Median time to ANC >1000/µL: 21d vs 19d vs 24d (p = NR)	None reported	*With sargramostim:*<br>• Decreased time to ANC >500/µL compared to no CSF
O'Day 1994 (2)	Phase 2;<br>Open label<br><br>Hematologic malignancy (N=72 lymphoma)	ABMT preparative regimen[c] then<br><br>Sargramostim 250 µg/m²/d continuous IV x 21d, started within 2h of marrow reinfusion (n=26)<br>vs<br>historic controls (n=46)	*Sargramostim vs control:*<br>• Median time to ANC ≥500/µL: 14d vs 22d (p = .0001)<br>• Median time to ANC ≥1,000/µL: 16d vs 27d (p < .0001)<br>• Median time to platelets ≥20,000/µL: 22d vs 19d (p = NS)<br>• Median time on IV antibiotics: 7d vs 13d (p = .0004)<br>• Median duration of hospitalizations: 24d vs 28d (p = .0003)	*TRAE sargramostim vs control:*<br>• Fever:<br>○ >101°F during transplant: 96% vs 83%<br>○ During agranulocytosis: 48% vs 95%<br>○ Persistent fever (ANC >500/µL), unrelated to infection: 72% vs 11%<br>• Rash: 42% vs 41%<br>• Serositis: 12% vs 0%<br>• Peripheral edema: 23% vs 4%<br>• Bone pain: 15% vs 0%	*With sargramostim:*<br>• Decreased time to ANC ≥500 and 1,000/µL<br>• Fewer antibiotic days<br>• Reduced length of hospital stay

Citation	Design/Patient Population	Treatment	Efficacy	Treatment Adverse Events Specific to Sargramostim or Comparator	Comment
Bunn 1995 (3)	Phase 3;<br>Randomized;<br>Open label<br>Multicenter<br><br>Solid malignancy (N=230 small cell lung cancer)	Chemo-radiotherapy[d] then:<br><br>No sargramostim<br>vs<br>Sargramostim 250 µg/m^2 SC BID d4-18 in 21d cycles x 6	*Sargramostim vs no sargramostim:*<br>• ANC <500/µL: 18% vs 24% (p = .01)<br>• Platelets <25,000/µL: 35% vs 6% (p < .001)	*Sargramostim vs no sargramostim:*<br>• Toxic deaths: n = 9 (n = 5 pulmonary) vs n= 1 (pulmonary) (p < .01)<br>• Grade ≥3 non-hematologic TEAE higher with sargramostim:<br>o Infection (p = .04)<br>o Respiratory infection (p < .001)<br>o Dyspnea (p < .01)	*Sargramostim vs no sargramostim:*<br>• Increased thrombocytopenia<br>• Increased toxic deaths and non-hematologic AE<br>• Avoid sargramostim prophylaxis in small cell lung cancer therapy with cisplatin, etoposide and thoracic radiation
Jones 1996 (4)	Phase 3;<br>Randomized;<br>Double-blind;<br>Placebo-controlled<br><br>Solid malignancy (N=142 breast cancer)	Chemotherapy[e] then:<br><br>Placebo<br>vs<br>Sargramostim 250 µg/m^2 SC d3-15 in 21d cycles x 4	*Sargramostim vs placebo:*<br>• Median days ANC <500/µL: 2.8d vs 6.8d (p < .001)<br>• Median days ANC <1,000/µL: 6.0d vs 9.1d (p < .001)	*TEAE sargramostim vs placebo:*<br><br>• Late-cycle grade 4 thrombocytopenia (cycles 3 and 4) in GM-CSF recipients<br>• Grade 1-2 injection site reactions 53% vs 14%<br>• Low-grade fever: 51% vs 26%<br>• No difference in any Grade 3-4 nonhematologic TEAE	• GM-CSF use associated with significant increase in FAC dose-intensity as chemotherapy delivered on time more often in GM-CSF group (p < .001 for each drug)<br>• Decreased days of ANC <500 and 1,000/µL with sargramostim
O'Shaughnessy 1996 (5)	Phase 3;<br>Open label;<br>Randomized<br><br>Solid malignancy (N=53 breast cancer)	FLAC[f];<br>starting d4 until ANC >5,000/µL and platelets >100,000/µL:<br>Sargramostim 250 µg/m^2/day SC<br>vs<br>PIXY321[g] 375 µg/m^2 BID SC	*Sargramostim vs PIXY321:*<br>• Mean days ANC <500/µL: 4.5d vs 5.6d (p = NS)<br>• Mean days ANC <1000/µL: 7d vs 8.3d (p = .015)	*TRAE with sargramostim:*<br>• Grade 3 fatigue (n = 1)<br><br>*Sargramostim vs PIXY321:*<br>Grade 2-3 local skin reactions: 6 vs 21 (p < .001)	Fewer days ANC <1,000/µL and fewer skin reactions with sargramostim vs PIXY321

Citation	Design/Patient Population	Treatment	Efficacy	Treatment Adverse Events Specific to Sargramostim or Comparator	Comment
Yau 1996 (6)	Phase 3<br>Randomized;<br>Double-blind;<br>Placebo-controlled;<br>Multicenter<br><br>Hematologic (n=10 lymphoma) and solid malignancy (n=46 breast cancer)	Chemotherapy[h] then:<br><br>Placebo<br>vs<br>Sargramostim 250 µg/m^2/d SC BID d4 until ANC >1,500/µL x 2d (or maximum 49d)	*Sargramostim vs Placebo (cycle 1):*<br>• Median days ANC<500/µL 10d vs 12d (p = .01)<br>• Median days ANC <1,000/µL 10d vs 13d (p = .004)	No differences in grade 3-4 nonhematologic TEAEs	Sargramostim use decreased days ANC <500/µL and 1,000/µL
Beveridge 1997 (7)	Phase 3<br>Randomized;<br>Double-blind;<br>Multicenter<br><br>Hematologic (n=27; lymphoma n=23);<br>Solid malignancy (n=84; breast cancer n=58)	Sargramostim 300 µg (193 µg/m^2) (n=75)<br>vs<br>G-CSF 481 µg (7 µg/kg) (n=62)<br><br>SC QD x 5-7d	Not reported	*Sargramostim vs G-CSF:*<br>• Grade 1 fever: 48% vs 26% (p = .01)<br>• No difference in grade 3 systemic or local adverse events	No statistically significant differences in incidence or severity of local or systemic AEs possibly related to sargramostim vs G-CSF
Beveridge 1998 (8)	Phase 3:<br>Randomized;<br>Double-blind;<br>Multicenter<br><br>Hematologic (n=50; lymphoma); Solid malignancy (n=90; 50% breast, 32% lung), and other malignancy (n=41)	Sargramostim 250 µg/m^2 SC daily (n=79)<br>vs<br>G-CSF 5 µg/kg SC daily (n=102)	*Sargramostim vs G-CSF:*<br>• Mean time to ANC >1500/µL: 5.7d vs 4.6d (p = .0001)<br>• Mean time to ANC >1000/µL: 5.1d vs 4.5d (p = .009)<br>• Mean time to ANC >500/µL: 3.3d vs 3.6d (p = NS)	*TRAEs of sargramostim vs G-CSF:*<br>• Chills: 1.2% vs 1.9% (p = NS)<br>• Grade 2 Fever, 4-hr post injection: 3.8% vs 10.7% (p = NS)	No difference in time to ANC >500/µL but increased time to ANC >1,000 and 1,500/µL with sargramostim
Schiller 1998 (9)	Phase 2;<br>Multicenter; Single arm<br><br>Hematologic malignancy (N=55 multiple myeloma)	Autologous CD34$^+$-selected PBSCT<br>plus<br>Sargramostim 500 µg QD IV/SC until ANC >1,000/µL x 3d	*Sargramostim:*<br>• Median time to ANC >500/mL: 12d<br>• Median time to platelets>20,000/mL: 12d	None reported	Effective use of sargramostim with autologous CD34$^+$-selected PBSCT

Citation	Design/Patient Population	Treatment	Efficacy	Treatment Adverse Events Specific to Sargramostim or Comparator	Comment
Steward 1998 (10)	Phase 3<br>Randomized;<br>Double-blind;<br>Placebo-controlled;<br>Multicenter<br><br>Solid malignancy (N=300 small cell lung cancer)	Intensified chemotherapy[i] every 3 wk x 6 cycles<br>vs<br>Standard chemotherapy[i] every 4 wk x 6 cycles<br><br>Second randomization:<br>Placebo<br>vs<br>Sargramostim 250 µg/m^2 SC daily x 14d between cycles	*Sargramostim vs placebo:*<br>• Incidence febrile neutropenia:<br>○ Intensified therapy: 56% vs 55%<br>○ Standard therapy: 53% vs 51%	*TRAE sargramostim vs placebo:*<br>• Injection-site reactions: 5% vs 0%<br>• Arthralgia/myalgias: 1.3% vs 1.3%	Sargramostim use did not significantly decrease time to hematopoietic recovery or incidence of chemotherapy-related adverse events
Le Cesne 2000 (11)	Phase 3<br>Randomized;<br>Multicenter<br><br>Solid malignancy (N=314, soft tissue sarcoma)	Standard dose chemotherapy[j] every 3 wk<br>vs<br>High-dose chemotherapy[j] + sargramostim 250 µg/m^2 SC d3-16 or until ANC >1,000/µL every 3 wk	*Standard dose chemotherapy vs high-dose chemotherapy + sargramostim:*<br>• Median progression-free survival 19 wk vs 29 wk (p = .03)	*TEAE Standard dose chemotherapy vs high-dose chemotherapy + sargramostim:*<br>• Febrile neutropenia: 4.6% vs 16.6% (p = .0004)<br>• Grade 3-4 thrombocytopenia 8% vs 50%<br>• Grade 3-4 neutropenia 92% vs 90%<br>• Grade 3-4 asthenia 4.5% vs 16% (p = .0005)<br>• Grade 3-4 stomatitis 3.9% vs 13% (p = .008)	Sargramostim facilitated dose-intensity and improved progression-free survival but no difference in overall survival

[b]p value for sargramostim and G-CSF group vs no CSF group; [c]cyclophosphamide x 2 d + total body irradiation 1,200 cGy (200 cGy BID over 3 d); [d]cisplatin and etoposide d1-3 and thoracic radiation therapy 4,500 cGy (25 fractions); [e]fluorouracil, doxorubicin, and cyclophosphamide on d1; [f]FLAC on d1-3 in 21-day cycles x 5; [g]PIXY321 is an IL-3/GM-CSF fusion protein; [h]cyclophosphamide d1-2, etoposide d1-3, cisplatin d1-3 (maximum 3 cycles); [i] V-ICE (ifosfamide, carboplatin, and etoposide d1-2, then etoposide d3, vincristine d15); [j]doxorubicin and ifosfamide d1

Abbreviations: ABMT, autologous bone marrow transplant; AE, adverse event; ANC, absolute neutrophil count; BID, twice daily; CSF, colony-stimulating factor; FLAC, 5-fluorouracil, calcium leucovorin, doxorubicin and cyclophosphamide; G-CSF, granulocyte colony-stimulating factor; GM-CSF, granulocyte-macrophage colony-stimulating factor; ICE, ifosfamide, carboplatin, etoposide; NS, not significant; PBSCT, peripheral blood stem cell transplant; SC, subcutaneous; TEAE, treatment-emergent adverse event; TRAE, treatment-related adverse event; V-ICE, vincristine, ifosfamide, carboplatin, etoposide.

Supplementary References

1. Fields KK, Elfenbein GJ, Perkins JB, Janssen WE, Ballester OF, Hiemenz JW, et al. High-dose ifosfamide/carboplatin/etoposide: maximum tolerable doses, toxicities, and hematopoietic recovery after autologous stem cell reinfusion. *Semin Oncol* (1994) 21(5 Suppl 12):86-92.

2. O'Day SJ, Rabinowe SN, Neuberg D, Freedman AS, Soiffer RJ, Spector NA, et al. A phase II study of continuous infusion recombinant human granulocyte-macrophage colony-stimulating factor as an adjunct to autologous bone marrow transplantation for patients with non-Hodgkin's lymphoma in first remission. *Blood* (1994) 83(9):2707-14.

3. Bunn PA, Jr., Crowley J, Kelly K, Hazuka MB, Beasley K, Upchurch C, et al. Chemoradiotherapy with or without granulocyte-macrophage colony-stimulating factor in the treatment of limited-stage small-cell lung cancer: a prospective phase III randomized study of the Southwest Oncology Group. *J Clin Oncol* (1995) 13(7):1632-41. doi: 10.1200/JCO.1995.13.7.1632.

4. Jones SE, Schottstaedt MW, Duncan LA, Kirby RL, Good RH, Mennel RG, et al. Randomized double-blind prospective trial to evaluate the effects of sargramostim versus placebo in a moderate-dose fluorouracil, doxorubicin, and cyclophosphamide adjuvant chemotherapy program for stage II and III breast cancer. *J Clin Oncol* (1996) 14(11):2976-83. doi: 10.1200/JCO.1996.14.11.2976.

5. O'Shaughnessy JA, Tolcher A, Riseberg D, Venzon D, Zujewski J, Noone M, et al. Prospective, randomized trial of 5-fluorouracil, leucovorin, doxorubicin, and cyclophosphamide chemotherapy in combination with the interleukin-3/granulocyte-macrophage colony-stimulating factor (GM-CSF) fusion protein (PIXY321) versus GM-CSF in patients with advanced breast cancer. *Blood* (1996) 87(6):2205-11.

6. Yau JC, Neidhart JA, Triozzi P, Verma S, Nemunaitis J, Quick DP, et al. Randomized placebo-controlled trial of granulocyte-macrophage colony-stimulating-factor support for dose-intensive cyclophosphamide, etoposide, and cisplatin. *Am J Hematol* (1996) 51(4):289-95. doi: 10.1002/(SICI)1096-8652(199604)51:4<289::AID-AJH7>3.0.CO;2-S.

7. Beveridge RA, Miller JA, Kales AN, Binder RA, Robert NJ, Heisrath-Evans J, et al. Randomized trial comparing the tolerability of sargramostim (yeast-derived RhuGM-CSF) and filgrastim (bacteria-derived RhuG-CSF) in cancer patients receiving myelosuppressive chemotherapy. *Support Care Cancer* (1997) 5(4):289-98.

8. Beveridge RA, Miller JA, Kales AN, Binder RA, Robert NJ, Harvey JH, et al. A comparison of efficacy of sargramostim (yeast-derived RhuGM-CSF) and filgrastim (bacteria-derived RhuG-CSF) in the therapeutic setting of chemotherapy-induced myelosuppression. *Cancer Invest* (1998) 16(6):366-73. doi: 10.3109/07357909809115775.

9. Schiller G, Vescio R, Freytes C, Spitzer G, Lee M, Wu CH, et al. Autologous CD34-selected blood progenitor cell transplants for patients with advanced multiple myeloma. *Bone Marrow Transplant* (1998) 21(2):141-5. doi: 10.1038/sj.bmt.1701055.

10. Steward WP, von Pawel J, Gatzemeier U, Woll P, Thatcher N, Koschel G, et al. Effects of granulocyte-macrophage colony-stimulating factor and dose intensification of V-ICE chemotherapy in small-cell lung cancer: a prospective randomized study of 300 patients. *J Clin Oncol* (1998) 16(2):642-50. doi: 10.1200/JCO.1998.16.2.642.

11. Le Cesne A, Judson I, Crowther D, Rodenhuis S, Keizer HJ, Van Hoesel Q, et al. Randomized phase III study comparing conventional-dose doxorubicin plus ifosfamide versus high-dose doxorubicin plus ifosfamide plus recombinant human granulocyte-macrophage colony-stimulating factor in advanced soft tissue sarcomas: A trial of the European Organization for Research and Treatment of Cancer/Soft Tissue and Bone Sarcoma Group. *J Clin Oncol* (2000) 18(14):2676-84. doi: 10.1200/JCO.2000.18.14.2676.
