# Supplementary material for: Sargramostim (rhu GM-CSF) as Cancer Therapy (Systematic Review) and An Immunomodulator. A Drug Before Its Time?
Source: Front Immunol. 2021 Aug 17;12:706186. doi: 10.3389/fimmu.2021.706186 (PMC8416151; doi:10.3389/fimmu.2021.706186)
Supplement: Supplementary file 2 [file Table_2.pdf]

Supplementary Material

Table 2. Initial studies supporting approval of indications for sargramostim in hematopoietic recovery

Citation	Model	Disease Target	Study Design	Treatment	Treatment Adverse Events	Outcomes	Comments
**Treatment of Delayed Neutrophil Recovery After Allogeneic or Autologous BMT**							
Nemunaitis 1990 (1)	Allogeneic & autologous BMT	Hematologic (n=31) and solid (n=4) malignancy; aplastic anemia (n=2)	Phase 1-2<br><br>n = 192	BMT then: Sargramostim 60–1,000 µg/m^2/d IV over 2h x 14d/21d (n=37)<br>vs<br>Historic controls with graft failure (n=155)	*TRAE Sargramostim ≥500 µg/m^2/d (n = 4; 6 courses):*<br>• n = 4, myalgias and bone pain during infusion<br><br>*TRAE Sargramostim ≤250 µg/m^2 (n = 33; 46 courses):*<br>• n = 1, sternal/joint pain	*Sargramostim:*<br>• ANC >500/µL (within 14d of the final course): 57%<br><br>*Sargramostim vs historic control:*<br>• OS prolonged with sargramostim (p = .001)<br>• Infection-related death: 21% vs 59% (p = NR)	Improved survival with sargramostim; majority of patients with neutrophil recovery by day 14 along with resolution of fever and infection
**Acceleration of Myeloid Reconstitution After Autologous BMT**							
Nemunaitis 1991 (2) / Rabinowe 1993 (3)	Autologous BMT	Hematologic malignancy (N=128)	Phase 3; Multicenter; Randomized; Double-blind; Placebo-controlled	BMT then: Sargramostim 250 µg/m^2 IV over 2 hr daily x 21 d (n=65)<br>vs<br>Placebo (n=63)	No differences between groups	*Sargramostim vs placebo:*<br>• Median time to ANC ≥500/µL: 19d vs 26d (p < .001)<br>• Median time to ANC >1000/µL: 26d vs 33d (p = .009)<br>• ANC >500/µL within 21d: 59% vs 32% (p < .002)	• Decreased time to ANC >500 and 1000/µL, IV antibiotic use, and hospital LOS with sargramostim<br>• On follow-up (median of 36 month), no graft failure, no increased risk of leukemogenesis<br>• Sargramostim treatment an independent predictor of accelerated neutrophil engraftment regardless of disease or number/type of prior therapies

Citation	Model	Disease Target	Study Design	Treatment	Treatment Adverse Events	Outcomes	Comments
**Acceleration of Myeloid Reconstitution After Allogeneic BMT**							
Nemunaitis 1995 (4)	Allogeneic BMT	Hematologic malignancy (n=102); aplastic anemia (n=7)	Phase 3; Multicenter; Randomized; Double-blind; Placebo-controlled	BMT then: Sargramostim 250 µg/m² IV over 4h d0-d20 (n=53) vs Placebo (n=56)	No differences between groups	*Sargramostim vs placebo:*<br>• Median time to ANC ≥500/µL: 13d vs 17d (p = .0001)<br>• Median time to ANC ≥1,000/µL: 14d vs 19d (p = .0001)<br>• Median time to platelets ≥20,000/µL: 24d vs 26d (p = NS)	Decreased time to ANC ≥500 and 1000/µL, infection, bacteremia, mucositis, LOS with sargramostim
**Reduced Time to Neutrophil Recovery and Fewer Infections After Acute Myeloid Leukemia Induction (≥55 years old)**							
Rowe 1995 (5) / Rowe 1996 (6)	Chemotherapy induction	Hematologic malignancy (N=124)	Phase 3; Randomized; Double-blind; Placebo-controlled; Multicenter	Induction and consolidation chemotherapy[a] then: Sargramostim 250 µg/m² IV over 4 hr daily starting d11 of induction and consolidation until ANC ≥1,500 x3d or a maximum of 42d (n=62) vs Placebo (n=62)	Grade 3-4 hemorrhagic, hepatic and neurologic adverse events were reduced in the subjects given sargramostim	*Sargramostim vs placebo:*<br>• Median time to ANC >500/µL: 13d vs 17d (p = .001)<br>• Median time to ANC >1000/µL: 14d vs 21d (p = .001)<br>• Median time to platelets >20,000/µL: 11d vs 12d (p = NS)<br>• Median OS: 10.6 mo vs 4.8 mo (p = .048)<br>• Death from infection: 6% vs 23% (p = .019)<br>• Death from fungal infection (overall): 2% vs 19% (p = .006)<br>• Death from fungal infection (grade 3/4): 13% vs 75% (p = .02)<br>• Death related to pneumonia: 14% vs 54% (p = .046)	• Sargramostim used in patients with *de novo* AML (age 55-70 yr) decreased time to ANC >500 and 1000/µL, reduced infections, death from infection, death from fungal infection, death from pneumonias<br>• Increased OS with sargramostim

Citation	Model	Disease Target	Study Design	Treatment	Treatment Adverse Events	Outcomes	Comments
**Increased Survival in Hematopoietic Acute Radiation Syndrome**							
Clayton 2021 (7)	Nonhuman primate	Myeloablation (N=108)	Randomized; Double-blind; Placebo-controlled	$LD_{50\text{-}60/60}$ TBI or $LD_{70\text{-}80//60}$ TBI<br><br>Starting 48h post irradiation (without blood transfusions or individualized antibiotics): Sargramostim 7 µg/kg/d SC (~250 µg/m^2/d) vs vehicle<br><br>SC daily until ANC >1,000/µL x 3d or ANC ≥10,000/µL	Not reported	*Sargramostim vs vehicle:*<br>• Survival (day 60):<br>o $LD_{50\text{-}60/60}$: 78% vs 42% (*p* = .0018)<br>o $LD_{70\text{-}80//60}$: 61% vs 17% (*p* = .0076)<br>• Infections:<br>o $LD_{50\text{-}60/60}$: 32% vs 63% (*p* = .0001)<br>o $LD_{70\text{-}80//60}$: 37% vs 84% (*p* < .0001)	Increased survival with sargramostim in the absence of intensive supportive care

[a]Induction with daunorubicin d1-3, cytarabine d1-7 x 2 (maximum) cycles plus consolidation with cytarabine

Abbreviations: AML, acute myeloid leukemia; ANC, absolute neutrophil count; BMT, bone marrow transplantation; IV, intravenous; $LD_{50\text{-}60/60}$, dose lethal in 50-60% by D60; $LD_{70\text{-}80/60}$, dose lethal in 70-80% by D60; LOS, length of stay; NR, not reported; NS, non-significant; OS, overall survival; SC, subcutaneously; TBI, total body irradiation; TRAE, treatment-related adverse event.

Supplementary References

1. Nemunaitis J, Singer JW, Buckner CD, Durnam D, Epstein C, Hill R, et al. Use of recombinant human granulocyte-macrophage colony-stimulating factor in graft failure after bone marrow transplantation. *Blood* (1990) 76(1):245-53.

2. Nemunaitis J, Rabinowe SN, Singer JW, Bierman PJ, Vose JM, Freedman AS, et al. Recombinant granulocyte-macrophage colony-stimulating factor after autologous bone marrow transplantation for lymphoid cancer. *N Engl J Med* (1991) 324(25):1773-8. doi: 10.1056/NEJM199106203242504.

3. Rabinowe SN, Neuberg D, Bierman PJ, Vose JM, Nemunaitis J, Singer JW, et al. Long-term follow-up of a phase III study of recombinant human granulocyte-macrophage colony-stimulating factor after autologous bone marrow transplantation for lymphoid malignancies. *Blood* (1993) 81(7):1903-8.

4. Nemunaitis J, Rosenfeld CS, Ash R, Freedman MH, Deeg HJ, Appelbaum F, et al. Phase III randomized, double-blind placebo-controlled trial of rhGM-CSF following allogeneic bone marrow transplantation. *Bone Marrow Transplant* (1995) 15(6):949-54.

5. Rowe JM, Andersen JW, Mazza JJ, Bennett JM, Paietta E, Hayes FA, et al. A randomized placebo-controlled phase III study of granulocyte-macrophage colony-stimulating factor in adult patients (> 55 to 70 years of age) with acute myelogenous leukemia: a study of the Eastern Cooperative Oncology Group (E1490). *Blood* (1995) 86(2):457-62.

6. Rowe JM, Rubin A, Mazza JJ et al. "Incidence of infections in adult patients (>55 years) with acute myeloid leukemia treated with yeast-derived GM-CSF (sargramostim): results of a double-blind prospective study by the Eastern Cooperative Oncology Group," in: Acute Leukemias V: Experimental Approaches and Management of Refractory Disease, eds. Hiddeman W, Buchner T, Wormann B, Schellong L, Ritter J, Creutzig U (Berlin, Germany: Springer-Verlag) (1996). 178-184.

7. Clayton NP, Khan-Malek RC, Dangler CA, Zhang D, Ascah A, Gains M, et al. Sargramostim (rhu GM-CSF) Improves Survival of Non-Human Primates with Severe Bone Marrow Suppression after Acute, High-Dose, Whole-Body Irradiation. *Radiat Res* (2021) 195(2):191-9. doi: 10.1667/RADE-20-00131.1.
